# Future Protein Supply and Demand: Strategies and Factors Influencing a Sustainable Equilibrium

**DOI:** 10.3390/foods6070053

**Published:** 2017-07-20

**Authors:** Maeve Henchion, Maria Hayes, Anne Maria Mullen, Mark Fenelon, Brijesh Tiwari

**Affiliations:** 1 Department Agri-Food Business and Spatial Analysis, Rural Economy and Development Programme, Teagasc Food Research Centre, Ashtown, Dublin D15 KN3K, Ireland; 2 Food BioSciences Department, Teagasc Food Research Centre, Ashtown, Dublin D15 KN3K, Ireland; Maria.Hayes@teagasc.ie; 3 Food Quality and Sensory Science, Teagasc Food Research Centre, Ashtown, Dublin D15 KN3K, Ireland; Anne.Mullen@teagasc.ie; 4 Teagasc Food Research Programme, Teagasc Food Research Centres, Ashtown and Moorepark, Fermoy, Co. Cork P61 C996, Ireland; Mark.Fenelon@teagasc.ie; 5 Food Chemistry and Technology Department, Teagasc Food Research Centre, Ashtown, Dublin D15 KN3K, Ireland; Brijesh.Tiwari@teagasc.ie

**Keywords:** protein, novel protein, protein demand, in vitro meat, algae, insect, dairy, meat, vegetal, consumer

## Abstract

A growing global population, combined with factors such as changing socio-demographics, will place increased pressure on the world’s resources to provide not only more but also different types of food. Increased demand for animal-based protein in particular is expected to have a negative environmental impact, generating greenhouse gas emissions, requiring more water and more land. Addressing this “perfect storm” will necessitate more sustainable production of existing sources of protein as well as alternative sources for direct human consumption. This paper outlines some potential demand scenarios and provides an overview of selected existing and novel protein sources in terms of their potential to sustainably deliver protein for the future, considering drivers and challenges relating to nutritional, environmental, and technological and market/consumer domains. It concludes that different factors influence the potential of existing and novel sources. Existing protein sources are primarily hindered by their negative environmental impacts with some concerns around health. However, they offer social and economic benefits, and have a high level of consumer acceptance. Furthermore, recent research emphasizes the role of livestock as part of the solution to greenhouse gas emissions, and indicates that animal-based protein has an important role as part of a sustainable diet and as a contributor to food security. Novel proteins require the development of new value chains, and attention to issues such as production costs, food safety, scalability and consumer acceptance. Furthermore, positive environmental impacts cannot be assumed with novel protein sources and care must be taken to ensure that comparisons between novel and existing protein sources are valid. Greater alignment of political forces, and the involvement of wider stakeholders in a governance role, as well as development/commercialization role, is required to address both sources of protein and ensure food security.

## 1. Introduction

UN figures projecting global population growth of almost 50% since 2000 to 9.5 billion by 2050 [1] seem to be generally accepted. In addition to giving rise to an increased demand for food as a result of more mouths to feed, other changes, such as increased incomes and urbanization, will result in changes in consumption patterns. Thus, not only will the amount of food required change, the type of foods demanded, and their relative contribution to diets, will change. Projected demand for protein is of particular interest, with projections that the world demand for animal-derived protein will double by 2050 [2], resulting in concerns for sustainability and food security. In part, this is because it is generally accepted that animal-based foods produce higher levels of greenhouse gases (GHG) than plant-based foods and these are associated with climate change [3]. This is compounded by the fact that increased demand for animal-based protein is expected to intensify pressure on land due to the need to produce more animal feed. This in turn will increase the conversion of forests, wetlands and natural grasslands into agricultural lands, which in itself has negative consequences for GHG emissions, biodiversity and other important ecosystem services [4]. This paper looks at a number of possible scenarios relating to future protein demand for human consumption, discusses factors influencing this demand and outlines some strategies to respond to the expected increased demand. It considers implications and opportunities for current and novel/emerging animal and plant-based protein sources. It concludes by discussing implications for future research, the agri-food industry and related stakeholders.

## 2. Protein Demand

In addition to increased demand arising from population growth, increased demand for protein globally is driven by socio-economic changes such as rising incomes, increased urbanisation, and aging populations whereby the contribution of protein to healthy aging is increasingly recognised [5,6], and recognition of the role of protein in a healthy diet. Economic development and increased urbanization is leading to major transitions in population-level dietary patterns in low and middle income countries in particular, such that most of the global increases in demand for foods of animal origin are seen in developing countries [6]. Some forces, however, provide a countervailing force slowing demand in developed countries. Such factors include increased awareness of the impact of food production and consumption on the environment and on health. In the context of protein, the negative impact is mainly associated with animal-derived protein with reports that 12% of GHG emissions derive from livestock production and that 30% of human-induced terrestrial biodiversity loss can be attributed to animal production [2]. Land use is also a concern; for example, in the EU two thirds of total agricultural area is used for livestock production and around 75% of protein-rich animal feed is imported from South America using large tracts of land there also [2]. Health concerns arise with over-consumption of protein, particularly when linked with saturated fatty acids and over consumption of processed meats. Ethical issues about animal production could also stifle demand with a trend towards flexitarianism and initiatives aimed at reducing meat consumption evident in some markets. 

**Figure 1:**
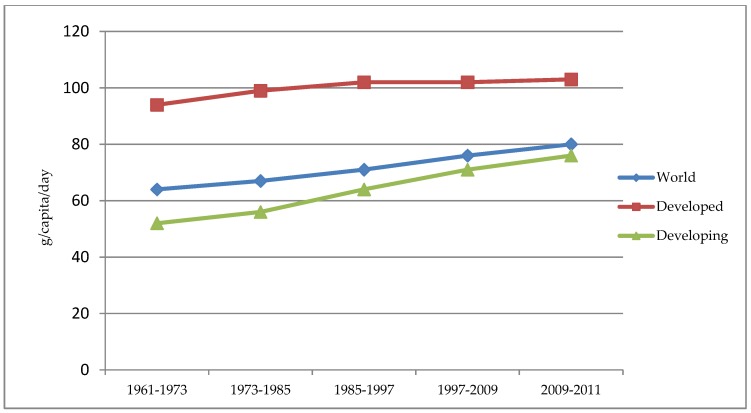
Evolution in protein consumption per capita (g/capita/day). Source: Author’s analysis based on food balance and population data obtained from http://faostat3.fao.org.

Figure 1 presents trends in protein consumption per capita between 1961 and 2011 for developed and developing countries. Differential levels are evident with developed countries showing higher absolute levels of consumption but developing countries showing significant growth rates over the period. Current protein demand for the 7.3 billion inhabitants of the world is approximately 202 million tonnes globally. However, even accepting a 2.3 billion growth in population, vastly different outcomes in terms of demand for protein result depending on assumptions made about average consumption for the future. Table 1 shows protein demand requirements for a range of possible scenarios. The scenarios presented are somewhat simplistic in that they are based on current knowledge about consumption levels in developed and developing countries as well as prevailing knowledge regarding the amount of protein required to meet basic nutritional needs. Developing detailed projections about future protein demand is beyond the scope of this paper. Nonetheless, the scenarios presented serve to illustrate the impact of variation in per capita consumption on projected demand, independent of population growth, and thus suggest the opportunity for demand side responses as a complement to supply side initiatives. Scenarios 1 to 3 assume maintenance of current consumption patterns for the existing population of 7.3 billion but have different assumptions for the additional 2.3 billion people that the UN project will exist. Scenario 1 assumes that the additional population will come from developing countries and that these consumers will have a per capita consumption level that is the same as current per capita consumption in developing countries. However, given the upward trajectory of demand in such countries, Scenario 3 assumes that per capita consumption for the additional population will be the same as for developed countries. Scenario 2 assumes an intermediate position with the additional population consuming the same level as the current global average. These scenarios show significant variation in additional demand, between 32% and 43% in these examples, depending on the level of per capita demand assumed. 

At least two other scenarios could be proposed based on current knowledge about protein consumption and nutrition. The first assumes the entire global population will consume protein at the highest rate at which it is currently being consumed by a population (103 g/hd/day) and the other assumes the entire global population will consume protein at a level which is considered to be adequate for the average sedentary adult. These scenarios show significantly different outcomes in future demand ranging from a 78% increase to a reduction of more than 10%.

Which scenario is most likely is dependent on the balance between competing forces that promote and restrict protein consumption. Given that most population growth is occurring in developing countries and that socio-economic factors in such countries are driving a nutrition transition towards higher intakes of protein, combined with evidence that the market for protein in developed countries continues to be driven by demographic and lifestyle changes, Scenario 5 is highly unlikely. Indeed, over a third of 18–34 years old in North America claim to be trying to consume as much protein as possible, whilst FAO research [8] highlights the staggering growth expected in demand for poultry in South East Asia (725% increase between 2000 and 2030), which is primarily attributed to increasing per capita consumption rates rather than increasing population levels. Furthermore, new research on the benefits of protein beyond muscle development and maintenance, for example research on the impact of protein on satiety and weight management [9] and more recently on hunger stimulation in hunger-suppressed individuals such as the elderly, continues to emerge, further stimulating increased demand. Combining these factors with enduring protein undernourishment [10], one is led to the conclusion that increased protein demand in excess of one third is highly likely for the future. This figure is conservative in comparison with figures from other sources, e.g., Swiss company Bühler who argue that an additional 50% will be needed by 2050 [11]. 

However, all proteins are not the same; they vary in terms of nutritional profile, digestibility and bioavailability, environmental implications, consumer acceptance, and other factors. Thus, future protein supply cannot be merely a matter of producing more of the same in the same proportions. Factors that influence the potential of various current and future protein supply sources are considered below. 

## 3. Existing Protein Sources

Currently vegetal sources of protein dominate protein supply globally (57%), with meat (18%), dairy (10%), fish and shellfish (6%) and other animal products (9%) making up the remainder [12]. 

### 3.1. Plant Based Protein (Cereals)

Cereal proteins account for the major portion of dietary protein intake globally [13,14] and are important for animals as well as humans. They are particularly important in the diet in developing nations and wheat accounts for largest group of plant protein sources in the Western diet [13]; in the form of bread, wheat is a key component of protein delivery in Europe with a typical loaf containing 8 g of protein per 100 g. Protein content ranges 10–15% with the largest protein content found in the storage proteins [14]. These storage proteins include the prolamins, globulins and germins [15]. Corn (maize), rice and wheat are the main staples consumed globally but, in some regions such as West Africa, millet is consumed extensively. In Southern India where protein malnutrition of infants is common, rice and millet are consumed regularly and, in Ethiopia, Teff, with an amino acid profile similar to egg protein, is preferred. Indeed, it has been calculated that a typical Ethiopian diet consists of 65 g of protein of which 41 g come from Teff while only 6 g come from animal protein consumption [16].

Maize (corn) is important for global food security. As a source of food (as opposed to feed), it accounts for 25% and 15% of total maize consumption in developing countries and globally, respectively. In addition, maize as a source of protein is very similar in terms of its contribution to calories intake globally. This ranges from 61% in Mesoamerica, 45% in Eastern and Southern Africa (ESA), 29% in the Andean region, 21% in West and Central Africa (WCA), to 4% in South Asia [17]. 

Compared to other cereals, the protein from oats is of high quality and the amino acid content and quality is comparable to soy protein [18]. Oat protein contains a higher content of the essential amino acid lysine compared to other cereals and has a lower proline and glutamic acid content. This attribute means that oat protein, once digested, can be tolerated by persons with gluten intolerance and allergies [19]. Rice does not contain large quantities of protein but rice protein flours have been prepared previously using enzymatic treatments with carbohydrate-hydrolysing enzymes to yield products with 91% protein [18,20].

While plant-based protein sources often lack one or more amino acids in sufficient quantity to meet human nutritional needs [21], combinations of different proteins, including cereal-pulse combinations, and supplementation, can help to overcome this in strict vegan or vegetarian diets. Cereals can also offer a significant health benefit as a rich source of bioactive peptides. A number of these are documented in the database BIOPEP [22]. Such food-derived bioactive peptides have gained increased interest as agents in the control of chronic diseases [22,23,24] and to reduce the risk of side effects arising from synthetic drugs usage. Bioactivities associated with cereal proteins include antioxidant, anti-inflammatory, cholesterol-lowering, satiety, anti-diabetic and others as reviewed recently [18]. They have a wide range of physiological effects that have been demonstrated in animal models previously. However, prolamins from some cereals, including wheat, barley, and rye, give rise to biologically-active anti-nutritional peptides following proteolysis that are able to adversely affect in vivo the intestinal mucosa of coeliac patients, whereas prolamins from other cereals such as maize and rice do not. Bioactivities associated with oat, barley and wheat protein derived peptides include opioid activity while rice protein contains the sequence RGD and lack celiac toxicity which could be beneficial in health and nutrition. Several groups have documented the use and potential of cereal derived bioactive peptides previously [18,25,26,27,28]. From an agronomical viewpoint, issues including cultivar use, environmental conditions and agricultural practices can affect the bioactive peptide content within cereal proteins. In terms of determining a physiological effect in humans, knowledge concerning the bioavailability of peptides and research on randomized clinical trials is necessary in order to understand the full potential of these proteins for health benefits and applications.

Notwithstanding the fact that plant-based protein is deemed to be preferable from a landuse and GHG emission perspective than animal-based proteins, plant protein is also subject to environmental criticisms. Such criticisms seem to be mainly related to the use of plant-based protein for animal feed and the increased environmental pressures associated with the development of industrialised farming systems to meet this demand. Soy, for example, is an important source of protein, however 85% of its production is used to feed animals and fish [29]. The drive to produce soy, to respond to the increased demand for animal protein, is associated with deforestation, and habitat loss, in South America in particular. There are ethical issues associated with this as around 12 million hectares of land outside Europe is required to produce protein-rich meal for European livestock production [10]. Other environmental issues such as water use, soil degradation, and pollution are also associated with high intensity plant protein production. The feeding of available edible plant protein to animals is itself a cause of controversy from an ethical perspective with previous authors arguing that if all such available edible plant protein were consumed by humans in an equitable manner then sufficient supplies of dietary protein would be available to feed the global population. The development of suitable vegetal protein products, which are accepted by consumers as preferred food items that compete with animal protein products rather than being positioned as imitations or extenders of animal protein products, is however required for a major shift to occur in the utilisation of plant-based protein.

### 3.2. Meat

Based on the FAO Food Balance Sheet data, it is clear that global meat consumption has increased significantly in recent decades. Analysis of such data by Henchion et al. [30] finds that overall meat consumption increased by almost 60% between 1990 and 2009. This trend is expected to continue, driven in particular by income growth in countries such as Asia, Latin America and the Middle East. Using 1997 as the base year, Rosegrant, Paiser, Meijer and Witcover [31] expect the quantity of meat demanded by consumers in developing countries to double by the year 2020. This growth is tempered somewhat by a suggestion that meat consumption per capita appears saturated in developed countries [32], compounded by aging populations, changing demographics and increased health and dietary awareness [30]. While the United Nations, some governments and several NGOs are implementing campaigns to reduce the amount of meat consumed [33,34], global meat consumption is expected to increase by 76% by 2050 [35]. This increased demand requires significant production growth. Given constraints on land and water availability and the impact of meat production on climate change, increased efficiencies in production practices and improvements throughout the wider food chain will be critical [36]. 

Meat is an important component of the human diet, and beef in particular has played a key role in food security in terms of providing energy, protein, and essential micronutrients. As highlighted by Gerber and colleagues [37], ruminants play a key role in converting fibrous material, which cannot be digested by humans, into protein which has a high nutritional value. Raw meat contains 20–25% protein depending on source and fat content, which, on loss of water due to cooking, can correspond to 28–36% in cooked meat. Protein from meat is an excellent source of essential amino acids and has high net protein utilization and digestibility [38]. In addition, meat is a key source of, often highly bioavailable, minerals (iron, zinc, and selenium) and vitamins (A, B9&12, D, and E), and has an ability, referred to as the “meat factor”, to enhance iron availability from other sources [39,40]. 

While meat consumption carries clear health benefits, over-consumption can lead to negative health impacts. The International Agency for Research on Cancer (IARC) report [41] suggested processed meat should be classified as “carcinogenic to humans” (every 50 g portion of processed meat increases risk of colorectal cancer by 18%). Red meat, on the other hand, was classified as “probably carcinogenic to humans” (every 100 g of red meat consumption leads to a 17% increased risk). While causation has not been fully elucidated, heme iron is thought to play a critical role through catalysis of N-nitroso-compounds, generation of lipid oxidation products and a possible direct cytotoxic effect. However, as discussed by De Smet and Voosen [42] “the benefits and risks associated with red and processed meat consumption should not necessarily cause dilemmas, if these meats are consumed in moderate amounts as part of balanced diets”. In relation to cardiovascular disease (CVD), however, research seems more inconsistent and there is debate regarding the paucity of data from randomized controlled trials (RCT). A recent meta-analysis of RCT supports the idea that consumption of under 0.5 servings, or 35 g, of total red meat per day does not negatively impact on CVD risk factors [43]. Public health concerns about livestock production are also relevant in relation to health, including zoonoses such as avian influenza, and concerns about emergence of novel diseases at animal-human-ecosystem interface.

From an environmental perspective, meat production, at a global level, contributes significantly to climate change and land use change [40]. High levels of greenhouse gases (GHG) are produced during meat production with ruminants contributing in a significant way. Land use, water, energy and chemical inputs (e.g., fertilizers) all reflect negatively on the environmental footprint from meat production. Of course, the relative level of impact varies depending on factors such as the species under consideration, production system (e.g., grass vs. concentrate production system), requirement for deforestation, and others. In particular, there is a case to be made for the promotion of meat produced from grass-fed ruminants [44].

In tandem to meat production, animals also produce a variety of other goods and services. Animals and cattle in particular, often serve as financial instruments and a route out of poverty in developing countries. Meat production is important for economic growth and poverty reduction in rural areas but needs to be managed carefully to ensure social effects are not negative.

Plant-derived alternatives to meat are being developed with many products already on the market. Quorn is the most well-known of these. It is derived from a fungus, using a natural fermentation process and wheat-derived glucose syrup. Quorn Foods, founded in 1985, currently sells about 22,000 tonnes of quorn in 16 countries, with investment underway to double production capacity in their UK plant. In vitro meat (see below) is presented as a longer-term alternative.

### 3.3. Dairy

Dairy based ingredients dominate the protein market, mainly because of a highly developed global milk industry and the fluid nature of milk that facilitates diversity through production of valuable by-product streams. They have functional properties and health benefits that are supported by scientific/medical studies, making them a suitable nutritional base for other foods. The global market for dairy protein is complex, multifaceted and driven by evolving markets and, more recently, trends in lifestyle nutrition. Protein ingredients are commonly manufactured as powder, but liquid-based convenience formats are growing rapidly. Developments in genetic selection coupled with feeding regimes of animals can be used to increase the protein content in milk, substantially increasing the yield of protein ingredients that can be manufactured. In Ireland for example, the protein content of milk has increased from an average of 3.2 to over 3.5% from 1996 to 2015. The development of membrane separation technologies such as micro- and ultra-filtration has created a diverse portfolio of protein ingredients, with tailored functional and/or nutritional attributes, generating multiple applications for a wide variety of foods across the globe. While global demand for protein is clear, predicting equilibrium between supply and demand for dairy protein is complicated by differences in rate at which intra- and inter-country milk production systems develop. India for example, with the highest milk production in the world, has a low input and low output system, comprising mainly small farms, and a large domestic market. A supply of high quality fluid milk, underpinned by quality control systems, is required to make viable any development in processing infrastructure (membrane, evaporation and drying equipment) for the manufacture of high value protein ingredients which could be traded globally. 

While skim and whole milk products contain protein, more concentrated systems can be categorized into:

(1) total milk protein: milk protein concentrate (MPC); isolate (MPI); hydrolysate (MPH);
(2) casein-based: caseinates from acid or rennet source (as sodium or calcium salts) and micellar casein; and
(3) whey-based: whey powder; demineralized whey powder (DWP); whey protein concentrate (WPC); whey protein isolate (WPI); whey protein hydrolysate (WPH).

Many of these ingredients are further sub-divided based on their protein content, e.g., WPC35 and WPC80 have 35 and 80% protein respectively. Developments in the manufacture of MPC have led to its increased usage for protein fortification, partly due to a low lactose content and acceptable flavor profile [45]. During production of dairy-based ingredients, as the protein content is increased, lactose and mineral salts are removed generating a by-product permeate stream, i.e., ultrafiltration of skim milk generates MPC and milk permeate whereas filtration of whey generates WPC and a whey permeate. The market for permeate powder is a key factor underpinning the business model for manufacture of a high protein concentrate, and the ability to valorise the entire milk or whey stream is a key determinant influencing export volumes of the various dairy protein powders. 

Many factors are driving the increased demand for dairy proteins, including the emergence of economies with increasing income. In countries like China, urbanization has provided new infrastructure capable of supporting distribution chains for dairy-based beverages. In Europe, the removal of quotas in 2015 resulted in a significant increase in milk production and protein ingredient manufacture has provided an avenue for utilization of this milk pool. The market for Greek-style yoghurt has expanded in the US, driven by the need for new consumer platforms for consumption of protein. In the US, liquid milk, ultra-filtrated to increase its protein content (50% more protein than regular milk), has been recently sold by Coca-Cola under the fairlife^®^ brand. The hugely successful protein bar segment has recently been extended to popular chocolate based bars, offering high protein (e.g., 18 g of protein per bar) alternatives, based on skim milk, milk protein and/or whey protein concentrate. Incorporation of protein into mainstream confectionary like this, adds to the complexity of protein markets, as now, global distribution chains are not solely associated with the sports and lifestyle/nutritional beverage sector. 

The global market for whey protein is continuing to grow based on increased demand for performance nutrition and beverages such as infant formula. Relatively new protein ingredients have been generated through microfiltration of milk to produce micellar casein protein and native whey. The global whey market is even further developed with individual fractions such as α-lactalbumin, β-lactoglobulin, Immunoglobulins (IgG), Lactoferrin, Lactoperoxidase and Glycomacropeptide (GMP) having application in infant nutrition, medical and sports beverages or foods. New research demonstrating the role of whey proteins in maintaining muscle mass (delayed development of sarcopenia) provides further opportunities for expansion of whey protein markets and is of particular relevance to an ageing population; these applications and those for special medical purposes often require high protein WPI (>80% protein) alternatives. Scientific studies have shown the beneficial effect of whey protein on muscle development [46,47] creating applications for body composition, sports, exercise and/or sarcopenia and even for serotonin synthesis in the brain [48]. The quality of the protein is a key attribute for such application; the emphasis is on individual, digestible amino acid content, which is excellent for dairy proteins, and in particular the whey protein α-lactalbumin, which also has high lysine availability [49]. 

One of the most important determinants of international trade in protein ingredients is safety, mainly through the impact of contamination and/or spoilage, where the distribution chain can be disrupted virtually overnight. An example of a food safety scandal was the adulteration of milk in China (in the media in 2008) with the compound melamine. Addition of melamine increases nitrogen levels and thereby falsifies protein content. The infant formula sector has particularly stringent quality specifications, including microbial specifications and chemical residues (e.g., veterinary drug residues). In addition, given the globally traded nature of dairy products, regulatory and political restrictions associated with biosecurity are of significance. 

Dairy production at farm level is subject to many of the negative environmental impacts associated with meat production due to its requirement for land and water and production of GHGs. However, grazing of dairy cows has been claimed to be preferable to grazing of suckler cows as they produce animal protein more efficiently [50]. Technological efficiency has already resulted in a significant reduction in the number of animals, and the quantity of food and water required to produce the same volume of milk. There has also been a significant reduction in manure, methane, N_2_O and the overall carbon footprint associated with milk produced currently compared to 1944 [51]. However, there is potential for significant improvement based on existing technologies with the significance of different strategies varying regionally and according to farming system. For example, in South Asian mixed dairy farming systems, feasible improvements in feed quality, animal health and husbandry could reduce GHG emissions by 38% of the base line emissions while for dairy mixed systems in OECD countries adoption of improved manure management systems, feed supplementation and energy saving equipment could reduce emissions by 14–17% of the baseline [52]. At the processing level, there is recognition of a growing need to adopt sustainable manufacturing processes, including energy and water/wastewater conservation; this is currently contributing to the re-engineering of unit operations within dairy manufacturing plants [53]. Environmental impact and sustainability of production during powder manufacture are key factors for milk processors when determining the type of protein ingredient to produce. A recent study by Finnegan et al., 2017 [54] applied environmental lifecycle assessment to estimate global warming potential (GWP) of manufacture of a range of dairy products. The authors used a system boundary covering the dairy, the farm, and transportation through to processing. They identified that during processing operations for powder production (SMP, WMP, and protein powders) the highest contribution to GWP was due to the energy intensive evaporation and drying steps. From an environmental perspective, dairy production/processing systems have been reviewed and the carbon footprint determined [54,55]. 

Country of origin labelling has become another key marketing tool for many countries and providing proof of provenance has become a prerequisite for purchase in some markets. A claim such as “Pasture based”, i.e., animals fed on grass outdoors, is an example of a trending marketable attribute associated with dairy products. Countries such as Ireland are leading the way in their approach to promoting a grass based feeding system, substantiating claims by scientific research, demonstrating the benefits of milk and its products produced by a grass fed animal [56,57]. 

Dairy based powders will remain a leading source of protein for the foreseeable future. The number of recombined food products will continue to grow in emerging economies and will drive demand. Nutritional applications for different stages of life from infants to elderly will develop in parallel with population and economic development. The ability to easily process liquid milk from bovine origin will ensure it continues to provide safe, high-quality products with good microbiological and functional properties. The rate of technological advancement in dairy will continue to evolve and support production of high quality ingredients that will facilitate supply to meet global demand for protein. 

## 4. New and Emerging Sources of Protein

### 4.1. Plant-Derived Protein (Pulses)

On a global basis, plant based protein is of immense importance and there is significant interest in its ability to meet growing demand for protein from non-meat sources. Thus, one of the key reasons for an increase in demand for plant-based protein is due to favourable comparisons with meat-based proteins. Plant-based protein is preferred to animal-based protein from an environmental perspective as it is associated with a lower land use requirement, and it is generally accepted that plant-based foods produce lower levels of greenhouse gases (GHG), which are associated with climate change, than animal-based foods [3]. Some plants offer unique advantages, e.g., pulses, being legumes, have the unique ability to fix nitrogen. Furthermore, due to the high cost and limited availability of animal proteins in several countries and consumer concerns over health benefits of animal-based proteins, increased attention has focused on the utilization of plant-based proteins as potential sources of low-cost dietary proteins for food use [58]. 

Among plants, pulses are considered an important source of dietary protein and other nutrients. In many regions of the world, pulses are the major source of protein in the diet, and often represent a necessary supplement to other protein sources. Indeed, pulses have a significant role in overcoming challenges associated with protein-energy malnutrition in developing and under-developed countries. For example, cowpea (*Vigna unguiculata*) is an important pulse grown and consumed in East and West African countries. Nutritionally pulses contain approximately 10% moisture, 21–25% crude protein, 1–1.5% lipids, 60–65% carbohydrates, and 2.5–4% ash. Chickpea is an exception as it contains about 4–5% lipids, and some pulses such as soy bean and lupin have been reported as having up to 45–50% protein. Proteins in pulses are found in the cotyledon and the embryonic axis of the seed with small amounts present in the seed coat. The cotyledons constitute the major portion of the pulse grain and hence make the highest contribution to protein content. Pulses like cereals show wide variation in protein content mainly due to genetic, environmental and agronomic factors. 

There are some concerns that vegans, or diet containing plant proteins as the major source of protein, are nutrient deficient due to unbalanced protein sources [59]. This is because, unlike animal proteins, plant proteins may not contain all the essential amino acids in the required proportions. Out of the nine essential amino acids (histidine, leucine, isoleucine, valine, threonine, methionine, phenylalanine, tryptophan and lysine) required by humans, pulses are deficient in sulphur containing amino acids (e.g., methionine). Pulses may also contain many anti-nutritional compounds such as hydrolase inhibitors and lectins which constitute part of the defensive mechanism of the seed; these may inhibit several biological functions [60,61]. Products obtained by using a blend of cereal and pulses can balance the amino acids and may improve the nutritive value of the product. Furthermore, fortification and food supplements mean it is easier nowadays to adopt a vegan or vegetarian dietary regime. Table 2 shows the level of proteins in selected pulses consumed around the world. 

Advances in production technologies, including technologies such as advanced monitoring systems (e.g., biomass monitoring and harvest monitoring) and remote sensing, and biological innovations in genomics and plant breeding will improve input efficiencies and help to anticipate and thus mitigate production risks (e.g., weather and pest related risks). New models of production are also emerging, e.g., soil-less growing and indoor farming. Production costs and energy requirements mean that such systems are currently confined to high value protected crops, e.g., salad crops, rather than field grown crops, with limited relevance for protein production currently. However, overall, in the foreseeable future, technological development will continue to position plant-based protein as a desirable option from a sustainability perspective.

### 4.2. Insect

Insect consumption (entomophagy), whereby eggs, larvae, pupae and adults of certain insects are consumed by humans, has occurred for thousands of years. Approximately 2000 species of insects have been used as food [71] and they are part of the traditional diets of at least 2 billion people [72], particularly in parts of Asia, Africa and South America where they provide important livelihood opportunities. Beetles are the most commonly consumed insects (31%), followed by caterpillars (18%); bees, wasps and ants (14%); grasshoppers, locusts and crickets (13%); cicadas, leafhoppers, planthoppers, scale insects and true bugs (10%); termites (3%); dragonflies (3); flies (2%); and others (5%) [72]. However, more recently, insects have been identified as an alternative source of protein for the Western world, not only as a delicacy or for emergency nutrition [73], supported by organizations such as the FAO [72] and the European Commission. Crickets, lesser mealworm and yellow mealworm are potential insects for application for food in the EU while black soldier fly, yellow mealworm and the common housefly have potential for use in feed products [13].

Proponents of entomophagy argue that it has a lower environmental impact compared to meat production [74]. Significantly, they argue that insects do not compete for land, require less water and emit lower levels of greenhouse gases and NH_3_ than regular livestock. They can be reared on organic side-streams thus creating value from and reducing waste products. However, the environmental impact of insect production is significantly influenced by insect’s diet, which in turn influences whether it can be used for food or feed purposes [75]. Another environmental advantage is that up to 80% of body weight is edible and digestible compared to 55% for chicken and 40% for cattle [35]. Being cold-blooded, they perform better in terms of feed conversion efficiency, and they reproduce more rapidly [76]. Many insects also have a favourable nutritional profile for humans, with most being highly digestible (77–98%), high in protein (crude protein 40–75% on a dry weight basis) [10] and a good source of essential amino acids, high in vitamins B_1_, B_2_ and B_3_ and the minerals iron and zinc [77]. However, many insects are deficient in certain amino acids, including tryptophan and lysine, and those with chitin exoskeletons have lower levels of digestibility. Insects vary considerably in fat, and thus energy levels, with some insects recorded as having 77 g/100 g dry weight. Some research argues that insects pose less risk of transmitting zoonotic diseases to humans, compared to animals and birds [72].

Most insects are, however, still collected from their natural environment (usually forests), which restricts supply according to season and location [78]. Furthermore, some researchers question insect consumption from a food safety perspective, arguing that the safety of insects as food is under-researched [77]. While insects are similar to other animal-derived products in that they are rich in nutrients and moisture (providing a medium for growth of unwanted microorganisms in certain conditions), the fact that insects are phylogenetically far removed from mammals, birds and some aquatic species regularly consumed as food means that significant differences are expected when making comparisons between them in terms of risks [77]. Furthermore, many insects are consumed whole, which includes their gut microflora which may affect microbiological quality of the food product [10] and specific health implications associated with using organic feedstocks to produce the insects need to be assessed (for example (undesirable) substrate materials can be transferred into the protein products and thus into the human food chain). 

Notwithstanding long traditions of domestication of bees and silk worms, and the practice of rearing insects for biological control, health and pollination [72] commercial insect farming for food purposes is only beginning to evolve, largely in proximity to concentrations of consumers. While insects are an established part of food culture in some countries and are eaten out of choice, there is evidence of some reluctance by Western consumers to accept insects as food as they are often considered as pests, and a source of contamination, and thus to be avoided. Van Huis and colleagues [72] relate the absence of a history of consuming insects to the difficulty of harvesting a proper meal of insects in temperate zones (insects tend to be less abundant, smaller and found less often in clumps in temperate zones). Nonetheless, food products are available on the market including cricket protein bars produced by British company Next Step Foods, and are on the shelves in European supermarkets including the Belgian supermarkets Delhaize and Carrefour. Widespread adoption of insects for food purposes will not however be achieved based on communicating their environmental and nutritional benefits. It will be dependent on addressing what Rozin and Fallon [79] identify as three important motives that lead to product rejection: negative sensory properties (distaste), harmful consequences (perceived danger) and “ideational” factors. Given that physical state is often used as a heuristic to evaluate food, processing insect protein to render it into an unrecognizable (e.g., as an ingredient in a familiar product) could be a productive strategy. Similarly, ensuring insects and insect-based products are acceptable from a sensory perspective and are safe to eat will be critical. 

The Belgian food safety authority allowed the sale of ten different types of insects for food consumption in 2013 following a federal ruling. However, the EU novel food regulation does not yet allow insects for human consumption to be sold. Regulatory changes are expected but it is not clear when this will happen, which means companies are currently focusing on insects for animal feed and pet food rather than human consumption. Commercial production of insects for human consumption will require the establishment of new value chains. Given that they are most likely to be accepted as ingredients rather than whole, such a chain includes ensuring a safe and reliable feedstock for the insect, mass rearing of insect larvae, their processing into insect ingredients, and application of these ingredients in final food products. All of these activities need to be developed simultaneously, resulting in costs and risks. Some organizations and commercial companies are setting up production on an industrial scale, e.g., the FAO established an insect farming project in the Philippines in 2010 [80], Swiss company Bühler is setting up pilot facility in China to process fly larvae and mealworms, and there are a few industrial scale enterprises in various stages of start-up within Europe for raising insects such as black soldier flies. The establishment of a trade group called International Platform for Insects for Food and Feed (IPIFF) is also significant. Nonetheless, current primary production systems remain expensive, with a need to develop automation processes to make insect production economically competitive; extraction processing systems are also too costly and need further development [72]. 

### 4.3. Algae

Marine plants such as seaweeds and microalgae represent a promising and novel future protein source. Both are collectively termed algae, however, seaweeds are complex multicellular organisms that grow in salt water or a marine environment, whereas microalgae are single celled organisms that can grow in a range of environmental conditions. Already €6.4 billion or 24 million tonnes of algae (mainly seaweed) are farmed globally [35] and protein-rich micro-algae are seen as a forerunner resource to close the so-called “protein gap”. Microalgae used for human consumption include *Arthrospira* spp. (*Spirulina* spp., namely, cyanobacteria), *Chlorella* spp. and *Dunaliella salina.* Only two microalgae are used primarily in the EU—*Spirulina* and *Chlorella* spp. Japan and China also supply these microalgae but there are some concerns regarding toxins, microbial load and other sanitary issues concerning their production. Microalgae are ordinarily produced in open, outdoor raceway ponds [81]. 

Nutritionally they are comparable to vegetable proteins but their development has been hampered by high production costs, technical difficulties in extraction and refining and sensory and palatability issues concerning their incorporation into food products. At present, microalgae are largely targeted for their EPA/DHA content and are sold as health foods, as cosmetics or as animal feed [82]. Approximately 30% of global algal production is sold for animal feed [13] with potential for further increases as dried, defatted algae could compete with soybean in pig and chicken feed, potentially replacing up to one third of soybean meal in their diets [31]. A US based company Solazyme is currently producing AlgaVia a whole algal product with 65% protein for sale as a food additive. They have two full-scale production lines in the US, with another in Brazil (capacity of 100,000 mt/year) [35]. 

Seaweeds can be harvested from the sea but are also increasingly cultivated. They, especially the red and green varieties, are very rich in protein (up to 47% dry weight in the red seaweed *Porphrya* sp.), are already considered as sea vegetables in countries such as France, and have consumer acceptance. The protein content of seaweeds differs according to species. Generally, the protein fraction of brown seaweeds is low (3 ± 15% of the dry weight) compared with that of the green or red seaweeds (10 ± 47% of the dry weight) [83]. The protein content of seaweeds also varies depending on the geographical location of harvest and the season of harvest. The amino acid content of seaweeds is comparable to protein sources such as eggs or soybean. The amino acids aspartic and glutamic acids constitute a large proportion of the amino acids of brown seaweeds (22–44% of total amino acids in some brown algal species). Studies carried out previously suggest that the method of extraction has an impact on digestibility of protein but that seaweed derived proteins are for the most part digestible and comparable to casein but the carbohydrate fraction and phlorotannins found in brown seaweeds can affect digestibility [83]. Some safety hazards include potential accumulation of heavy metals, high levels of iodine, and contaminants such as dioxins and pesticides [13].

Seaweeds could also be used in aquaculture diets or as animal feeds. A recent review by García-Vaquero [84] and others [83] suggest that the red seaweed *Porphyra* sp., has potential for use as feed for sea bream due to the high protein content and excellent amino acid profile of this species. Brown seaweeds including *Laminaria* sp., *Fucus* sp., and others, however, contain phlorotannins that can be viewed as anti-nutritional due to their protein binding capacity [85].

In addition to use in animal feed, seaweed protein and peptides can impart taste to foods or when used as food ingredients. Recently, a group in Oregon developed red seaweed, vegan bacon-tasting alternative to bacon. The taste of seaweeds is closely related to the fifth taste—umami. Umami taste is linked to the substance monosodium glutamate (MSG), the sodium salt of glutamic acid, an amino acid found in great abundance in seaweeds. Brown algae such as *Saccharina japonica* or *Laminaria jabonica* (common name is Konbu) have a particularly high MSG content which is released when the seaweed is heated or softened. In addition, red seaweeds including *Porphyra* sp, (common name Nori) also impart umami taste due to nucleic acids present within their cells. These compounds that cause umami taste in seaweeds are present due to the environment in which seaweeds grow which are often hostile. Seaweed ash salt was used in the past in regions where salt was not plentiful in countries such as Denmark and Brittany. Seaweed ash salt can impart a smoky taste to foods.

Regulatory restrictions on novel food and novel food ingredients, food safety, nutrition and food health claims can delay the pace of commercialization of algae [86]. Furthermore, the lack of publicly available economic and market information make it difficult to evaluate their industrial potential which is a hindrance in terms of seeking funding for research and development and for influencing policy.

## 5. Muscle protein sources

### 5.1. Fish

Seafood consumption is generally increasing in many parts of the World, particularly for coastal communities. In many places, fish and shellfish are the only readily available sources of protein that people can self-harvest, often throughout the year.

Aquaculture supplies 50% of all fish consumed globally today and by 2030 it is predicted that aquaculture will be the prime source of fish due firstly to demands from consumers, and secondly due to depletion of wild capture fisheries [87]. However, aquaculture needs to become more sustainable. Currently, it is over-dependent on a supply of fishmeal and fish oil, and there are concerns surrounding pollution and water quality, use of soy and chemicals in aquaculture feed, and habitat destruction. Negative social outcomes are also associated with aquaculture in countries where there are weak regulatory frameworks and there is concern about emerging diseases and disease transmission as a result of increased intensification and globalisation [35]. Companies such as Findus are however progressing towards more sustainable aquaculture systems, e.g., they now source shrimp farmed in Sweden that are produced using the “Biofloc” method [88]. This involves feeding shrimp excrement to microorganisms which in turn are consumed by the shrimp as a source of protein. Biofloc can produce up to 40 times more shrimp that conventional shrimp farming [88]. Thus, aquaculture has the potential to be a key element of a circular food system.

Reform of the European Common Fisheries Policy (CFP) in 2015 (Commission Delegated Regulation (EU) No. 1394/2014 further supports the marine sector being a key source of protein. The CFP aims to gradually eliminate the practice of discarding undersized fish as by-products at sea. Landing obligations will be phased in from 2015 to 2019 and will result in greater quantities of catch being landed at port. Undersized fish or under-utilised species such as Blue Whiting are not aesthetically pleasing to consumers and traditionally were thrown back to sea. However, there now exists an exciting opportunity for processors to convert this by-catch or marine “rest raw material” into value added ingredients due to the high protein and oil content of this by-catch. Several recent publications highlight the potential of this resource as a source of bioactive peptides [89], lipids [90] and small molecules/nutrients including vitamins and minerals [91]. 

Regular fish consumption is widely promoted as part of a healthy diet. There is strong scientific evidence for this. Fish have a high protein content compared to terrestrial animal meat and have a lower feed conversion rate (FCR) than land animals and more protein can be produced using this lower feed rate from fish [83]. Furthermore, fish protein is highly digestible and rich in essential amino acids that are limited in animal sourced protein including methionine (6.5% compared to 5.7% of total essential amino acids in fish compared to animal meat) and lysine (19.6% compared to 19.0% of total essential amino acids in fish compared to animal meat). Fish and shellfish consumption has several reported health effects including decreased risk of heart diseases, inflammation and arthritis. The health benefits of fish are mainly linked to the presence of long chain omega 3 (*n*-3) polyunsaturated fatty acids (PUFA) [83]. However, fish protein is also a rich source of bioactive peptides. These can have a myriad of positive health effects if consumed in appropriate concentrations and bioavailable within the human body. Health effects include control of blood pressure through inhibition of enzymes within the renin-angiotensin aldosterone system (RAAS), maintenance of bone health, control of inflammation (antioxidant peptides), mental health through the action of opioid peptides and platelet activating factor acetyl-hydrolase inhibitory (PAF-AH) peptides) and several other bioactivities [92].

While fish is seen as a healthy food by consumers, food safety risks such as heavy metal content could represent a potential barrier to consumption frequency, particularly concerning contamination of wild fish [89]. It is well known for example that the Baltic sea is heavily contaminated with heavy metals due to the presence of heavy industry in the landmass surrounding it that have used the sea as a “sink” for industrial waste for many years. Such chemical contamination, e.g., by heavy metals such as arsenic, mercury and cadmium and polychlorinated biphenyls (PCBs), can result in carcinogenic and toxicological impacts, as well as potentially mitigating the beneficial impact of omega 3 [93].

### 5.2. In Vitro Meat

In vitro meat, also referred to as cultured meat, cell-cultured meat or clean meat, is an animal product produced following cell isolation and identification, cell culture and tissue engineering protocols [94]. Several sources of cell can be used including embryonic stem cells from pre-implantation embryos or adult stem cells. Proponents argue that an in vitro meat bioreactor the size of a swimming pool could feed 40,000 people for a year, and that it would use 99% less land than the average for farmed beef [35]. The first cultured hamburger was created by Mark Post of Maastricht University in 2013, cost £200,000 and took two years to create [35]. Mosa Meat, a Dutch start-up, continues to develop it, focusing on improving flavor through co-culturing of fat cells, and cost reduction. Other companies, in the US and elsewhere, are also working towards similar goals. Current costs are estimated to be in the region of $11. Significant media attention and innovative research funding mechanisms, e.g., crowd funding, will support further technical development. 

In addition to economic and sensory factors, environmental, ethical and societal factors are at play. The possibility of producing meat without slaughtering animals carries an obvious benefit, as does the availability of a meat product with a much-reduced environmental impact. However, while on the positive side, ruminant greenhouse gases are lower by 96% [35], cultured meat will require more industrial energy than is required in livestock production. Furthermore, as cultured cells do not have an immune system, sterilization is a perquisite. Improvements in energy efficient processes may help circumvent this significant energy demand. The control afforded from the process means that consumers can be confident that they are being offered a standard product. However, the production process requires the use of chemicals e.g., hormones, nutrients, which while being of food grade, may not be attractive to consumer segments that value natural production systems. In a wider societal context, it is necessary to consider the impact on farmers’ livelihoods, in particular smaller operators and those in under-developed economies, who rely on livestock production for income and wealth. 

Technical challenges remain with the production of in vitro meat. Co-culturing of cells, e.g., muscle cells and adipose cells, and potential genetic instability due to speed of growth leading to cancerous cell formation, are two of particular note. The composition of the culture medium needs to meet the needs of growing cells and be considered safe for consumption. Further developments in industrial scale bioreactors are necessary. A big issue with the development of in vitro meat products is the complexity of factors which contribute to the final eating quality of real meat. While cell culture technology may be able to work towards providing the range of necessary micro and macro molecules which are reflective of raw muscle, mimicking the influence of environmental factors will remain very challenging. After slaughter, there are a series of events including glycolysis, calcium release, energy utilization, rigor onset, oxidation and denaturation, and enzyme effects, which all impact on the ultimate eating quality. These in turn are influenced by the environment in the abattoir such as chilling regime, hanging method and electrical inputs. Similarly, on-farm factors, in particular diet, can influence the flavour. When we consider meat production, while meat is the main product there are a wide variety of side products or co-products which are also produced. A systemic environmental analysis of in vitro meat is called for and must take this aspect into consideration [95].

Consumer attitudes will, obviously, have a great influence on the level of adoption of such a novel food. There is likely to be some public reluctance—a “yuck” factor—however in vitro meat offers some potential to respond to health-focused meat reducers and ethically motivated consumers who see environmental and animal welfare benefits. Interestingly, Verbeke et al. [96] suggest that vegetarians may not be the ideal primary target group for this product, given that the starting material has come from an animal. In a study of French consumers, Hocquette et al. [97] found that over half of the consumers surveyed accepted the feasibility of the concept but most were sceptical about it from a taste and health perspective, and only a minority would recommend or accept eating it instead of real meat. Taste tests with food critics in the UK actually reported that its taste was acceptable, albeit slightly bland. Willingness to try the new product but reluctance to incorporate into one’s daily diet was evident in US [98].

## 6. Discussion and Conclusions

Many scenarios are possible regarding future protein demand. While it is possible to adequately meet the nutritional requirements of a larger global population based on current supply volumes, it is highly likely the balance of driving and opposing forces, and a reluctance of policy makers to impose austere diets on citizens, will result in a world which will demand significantly more protein in the future. Furthermore, changing consumption patterns is a slow cultural process so, while demand size initiatives may be part of the solution in the longer term, for the short to medium term, more rapid changes are likely to originate from the supply side. While existing protein sources have been subject to various criticisms, and new concerns are emerging including the growth in antibiotic resistance associated with intensive animal production, they will have to continue to be significant sources of supply. This is for pragmatic reasons including the fact that they have established production and supply systems, are important economic sectors in rural areas and provide additional economic benefits in developing countries, their use is well established in consumers’ routines and practices and animal-based protein, in particular, has a significant role in diets, lifestyles and culture. However, it is also because we have not yet definitely established which diets are more sustainable; recent research makes a strong case that animal-based protein is an essential component of a sustainable diet. Van Kernebeek et al. [50] conclude that moderate consumption of meat is better for the environment compared to a vegan and vegetarian diet. Their results, which are based on a land use optimization model, contradict the results of previous life cycle assessment studies (which indicate that vegan diets use the least land followed by vegetarian diets) as “they do not consider the unsuitability of marginal lands to grow crops, the suitability of animals to use human-inedible products, and the co-production of meat and milk” [50] (p. 685). This leads them to argue that large populations can only be sustained if animal protein is consumed. Their contention is that land unsuitable for crop production is necessary to meet the dietary requirements of large populations and that this provides animal protein without competing with land for crops. A similar case is made by van Zanten et al. [4] when they assessed the land use efficiency of different livestock systems. They conclude that certain livestock systems can produce human digestible protein more efficiently than crops and that therefore livestock systems have a role in future food supply and contribute to food security. This research supports a case for the transformation of the food system, whereby livestock become vehicles to use resources (i.e., grass and food by-products) that cannot otherwise be used for food production, rather than being sources of high-quality protein [99]. Other recent research, linking GHG emissions to nutrition, further challenges conventional wisdom by indicating that dietary patterns with high levels of meat consumption do not necessarily result in the highest levels of GHG emissions [100]. 

Notwithstanding the argument to maintain existing protein sources in the diet, it is clear that the way we produce and consume protein has significant impacts on the environment and on human and animal health which necessitate action. Efforts have been underway since the 1960s to reduce the environmental impact of existing protein sources, particularly animal-based protein, in terms of GHG emissions, land, water and energy use, and biodiversity, as well as their social and public health impacts. Breeding, management, and nutrition efforts have led to significant increases in feed conversion efficiency, per-animal yields and decreases in GHG emissions per kg of animal product [92]. However there is significant potential for further efficiency gains because while technologies and practices that reduce emissions for example are in existence, they are not widely adopted despite most mitigation interventions offering economic as well as environmental benefits [37]. Such efficiency strategies are reported to have the potential to result in a reduction of up to 30% in GHG emissions from the livestock sector, if applied globally [99]. In this increased efficiency strategic perspective, the livestock sector (and its sectoral organizations) is identified as an important stakeholder in delivering on the mitigation efforts necessary to reduce GHG emissions and to improve its environmental footprint [37] with public policy to support on-farm investment and reduce risk associated with the adoption of new technologies, and extension efforts that disseminate best practice and highlight success stories, being important approaches.

As a complementary strategy, the UN, largely through the FAO, the EU and many NGOs, is driving the production of alternative proteins. Academics also are having an influential role. Halloran, Roos, Eilenberg, Cerutti and Bruun (2016) [101] identify the publication of the book Edible insects: Future prospects for food and feed security [72] as a “notable landmark” and state that it “captivated a multitude of stakeholders and successfully drew significant attention to the area” [72] (p. 2). However, as highlighted above, such proteins cannot be assumed to be safe or sustainable. They require thorough evaluation including holistic assessment, and appropriate regulatory frameworks. Their success will depend on “proving food safety, production costs, nutritional qualities, scalability and consumer acceptance” [35]. Consumer acceptance is a key hurdle and will require a significant shift for real growth to occur, whereby consumers incorporate such products into their habitual shopping behaviours as opposed to treating such products as novelties. Furthermore, their realization will require the development of new value chains and will require new initiatives to bring actors together who may not have traditionally worked together. An example of this is provided by the Forum for the Future, a project which brought the World Wide Fund for Nature (WWF), the Global Alliance for Improved Nutrition (GAIN), dairy firm Volac, flavour firm Firmenich, confectionery manufacturer Hershey and the meat-free brand Quorn together under the project “the Protein Challenge 2040”. This project aims to increase plant-based protein consumption, scale up sustainable feed and reduce protein waste. 

When comparing existing and novel sources of proteins and developing an overall strategy to address protein demand and thus to reflect prioritized sources, it is important that a balanced and holistic perspective is adopted as the story with respect to individual protein sources is equivocal. For example, while livestock production results in greenhouse gas emissions, livestock production can also result in a positive impact on biodiversity, e.g., through the maintenance of semi-natural grassland habitats that have high biodiversity levels [33] and nutrient recycling [10]. The same is true from a health perspective, e.g., while meat is an important source of nutrition and consuming meat has positive effects on health in developing countries [10], over-consumption of meat in developed countries has negative health impacts. The identification of appropriate comparison boundaries for novel and existing protein sources is also important as highlighted by Smetana et al. [75] in relation to insect-based and traditional products. These authors also cautioned against using results from one modelling scenario to approximate other models. Moreover, the value of many novel protein sources may extend beyond food. As highlighted above, microalgae have a value as ingredients for cosmetic, pharmaceutical and functional food applications as well as in polymer manufacture and in industry. Indeed, marine-derived bioactive peptide hydrolysates could return higher value than isolated proteins depending on their health benefits, which must be proven in accordance with the European Food Safety Authority legislation in Europe and the FDA in America and elsewhere. Quantifying their (higher) aggregate value may support more widespread adoption. Overall, this discussion points to the need for a change in the narrative from a discussion around “good” and “bad” sources of protein towards a better balance of sustainable protein [29]. 

There are many strategies are available to respond to growing global protein demand which span technological advancements, changes in the agri-food system and shifts in consumption patterns. Technology advancements include advances in data-enabled technologies as well as improvements in production and processing technologies. While there are concerns in some countries that the agrifood system is becoming more opaque [80], the future agri-food system “will need to play an active role in helping consumers make healthier and more sustainable food choices” [31]. Indeed, many stakeholders are needed to contribute to a “future food system which provides enough safe, authentic food for us all to have healthy lives now and in the future” [81]. Multi-stakeholder action is required at global level to be effective and fair [37]. Overall, this is quite a significant challenge, as, while it is clear that people need to start thinking globally about food, this is new and challenging and not something they are not yet used to doing (FSA). Active policy involvement is required to prevent irreversible consequences of dietary changes [5] and to ensure food security. 

While the drive towards diversified protein sources could be considered a threat by companies in meat and dairy sectors, for example, there are also significant opportunities for such companies to create new, high value by-products from undervalued waste streams as well as becoming directly involved in the development of new products from diversified protein sources. Policy makers have a key role to play in this regard, as well as in supporting opportunities to realize efficiencies and reduce food waste [35]. The WRAP report states that “ensuring the UK has a diversified, sustainable and healthy supply of protein will be one of the defining challenges of the coming decades”. This challenge is truly scalable, i.e., it will be one of the defining challenges for individual nation stages, regions and indeed the globe in the coming decades.

## Figures and Tables

**Table 1:** Impact of different consumption scenarios on annual protein demand ^1^.

Scenario	Pop. (000,000)	Consumption g/capita/day	Tonnes/Annum	% Change from 202.352 m Tonnes
1: Existing population at current consumption levels but increased population at average protein consumption for developing world for 2009–2011	9.6	76	263,802,000	+32%
2: Existing population at current consumption levels but increased population at average protein consumption for the world for 2009–2011	9.6	80	267,160,000	+33%
3: Existing population at current consumption levels but increased population at average protein consumption for the developed world for 2009–2011	9.6	103	286,468,500	+43%
4: Entire population at current max. consumption levels	9.6	103	360,912,000	+78%
5: Entire population at level required for sedentary adult	9.6	50 ^2^	175,200,000	−13%

Source: Authors’ calculations based on FAO/OECD data. ^1^ The data used measure “availability” as opposed to demand, i.e., demand is calculated as a residual based on “total available for human consumption = total food supply – feed – seed − industrial uses − waste”. While subject to several limitations as a measure of demand and likely to overestimate food consumption, in the absence of data from household surveys, it is accepted as a good proxy for consumption levels of a population as a whole and therefore for developing projections of future food supply needs [7]; ^2^ based on 2000 calories per day, 10% calorie intake as protein and 4 calories per gram of protein.

**Table 2:** Proximate composition of different pulse grains (g/100 g dry weight).

Pulses	Protein Content	Pulses	Protein Content
Kidney bean	23.58 [62,63]	Navy beans	22.33 [62]
Chickpea	19.30 [62,64], 19.29 [65]	Gt. northern bean	21.80 [62]
Lentils	25.80 [64], 26.1 [66]	French beans	18.81 [62]
Mung bean	23.86 [62], 27.5 [67]	Winged beans	29.65 [62]
Mungo bean	25.21 [62], 26.22 [65]	Hyacinth beans	23.90 [62]
Pigeon pea	21.70 [62,65]	White beans	23.36 [62,67]
Peas	24.55 [62], 19.3 [63]	Horse gram	22.50 [68,69,70]
Adzuki bean	19.87 [62,71]	Cowpea	23.85 [62,66], 24.1 [70]
Black beans	21.60 [62], 23.6 [64]	Navy beans	22.33 [62]
Lima beans	21.46 [62]	Gt. Northern bean	21.86 [62]
